# Evaluation of Safety and Probiotic Traits from a Comprehensive Genome-Based In Silico Analysis of *Ligilactobacillus salivarius* P1CEA3, Isolated from Pigs and Producer of Nisin S

**DOI:** 10.3390/foods13010107

**Published:** 2023-12-28

**Authors:** Ester Sevillano, Irene Lafuente, Nuria Peña, Luis M. Cintas, Estefanía Muñoz-Atienza, Pablo E. Hernández, Juan Borrero

**Affiliations:** Departamento de Nutrición y Ciencia de los Alimentos (NUTRYCIAL), Sección Departamental de Nutrición y Ciencia de los Alimentos (SD-NUTRYCIAL), Facultad de Veterinaria, Universidad Complutense de Madrid (UCM), Avenida Puerta de Hierro, s/n, 28040 Madrid, Spain; estsev01@ucm.es (E.S.); irelafue@ucm.es (I.L.); nuriapen@ucm.es (N.P.); lcintas@ucm.es (L.M.C.); ematienza@ucm.es (E.M.-A.); ehernan@ucm.es (P.E.H.)

**Keywords:** probiotic, *Ligilactobacillus*, bacteriocin, nisin S, megaplasmid

## Abstract

*Ligilactobacillus salivarius* is an important member of the porcine gastrointestinal tract (GIT). Some *L. salivarius* strains are considered to have a beneficial effect on the host by exerting different probiotic properties, including the production of antimicrobial peptides which help maintain a healthy gut microbiota. *L. salivarius* P1CEA3, a porcine isolated strain, was first selected and identified by its antimicrobial activity against a broad range of pathogenic bacteria due to the production of the novel bacteriocin nisin S. The assembled *L. salivarius* P1CEA3 genome includes a circular chromosome, a megaplasmid (pMP1CEA3) encoding the nisin S gene cluster, and two small plasmids. A comprehensive genome-based in silico analysis of the *L. salivarius* P1CEA3 genome reveals the presence of genes related to probiotic features such as bacteriocin synthesis, regulation and production, adhesion and aggregation, the production of lactic acid, amino acids metabolism, vitamin biosynthesis, and tolerance to temperature, acid, bile salts and osmotic and oxidative stress. Furthermore, the strain is absent of risk-related genes for acquired antibiotic resistance traits, virulence factors, toxic metabolites and detrimental metabolic or enzymatic activities. Resistance to common antibiotics and gelatinase and hemolytic activities have been discarded by in vitro experiments. This study identifies several probiotic and safety traits of *L. salivarius* P1CEA3 and suggests its potential as a promising probiotic in swine production.

## 1. Introduction

The overuse and misuse of antibiotics in both human and animal settings have contributed to the emergence and spread of antibiotic-resistant bacteria. Multidrug-resistant bacteria is a growing global problem that affects not only human health but also animal health and the environment, generating a significant negative impact on animal production [1]. The food and livestock industry have been working together for years in the development of different strategies aimed at reducing the use of antibiotics in animal production [2]. This has prompted the search for alternative antimicrobial strategies, including the exploration of bacteriocins which are promising candidates for combating antibiotic resistance [3,4]. Bacteriocins are antimicrobial peptides produced by bacteria as a defense mechanism against other bacteria. Unlike conventional antibiotics, bacteriocins often exhibit a narrow spectrum of activity, selectively targeting pathogenic or closely related bacteria while sparing beneficial microorganisms [3,5]. This selective targeting can help minimize disruption to the natural microbiota and reduce the potential for the development of resistance [6]. Moreover, bacteriocins have shown several advantages over antibiotics in terms of stability and safety. Many bacteriocins are heat-stable and retain their antimicrobial activity under various environmental conditions, including high temperatures and extreme pH levels. Additionally, bacteriocins are generally non-toxic to eukaryotic cells, making them potentially safe for therapeutic applications against antibiotic-resistant bacteria, including vancomycin-resistant *Enterococcus* (VRE) and methicillin-resistant *Staphylococcus aureus* (MRSA) [7].

The use of probiotic lactic acid bacteria (LAB) producing bacteriocins is considered as an effective, safe and economically profitable strategy to control bacterial infections and decrease the use of antibiotics within animal production [8]. The genus *Lactobacillus* holds significant importance within the LAB group, playing a vital role in promoting gastrointestinal health in various hosts, including pigs. The gastrointestinal tract (GIT) of pigs is a complex ecosystem hosting a diverse microbial community, which significantly influences the overall health and performance of these animals. These bacteria are considered an integral part of the porcine microbiota and contribute to the overall health and well-being of the pigs [9].

*Lactobacillus*, a formerly extensive genus, underwent a significant taxonomic reclassification that resulted in the division of *Lactobacillus* into 25 distinct new genera [10]. Consequently, this taxonomic update has drawn attention to numerous *Lactobacillus* species that possess remarkable degradative, transformative, and/or biosynthetic capabilities, thereby fueling widespread biotechnological interest in these organisms [11,12]. Certain *Lactobacillus* species have also been suggested as probiotics due to their ability to bestow health benefits upon the host when consumed in sufficient quantities [13]. *Lactobacillus* strains, including *Ligilactobacillus salivarius,* colonize the pig’s GIT, thereby exerting a range of beneficial effects including modulation of the gut microbiota, enhancement of immune responses, and maintenance of the gut barrier function. As a result, feed supplementation with *Ligilactobacillus*, particularly *L. salivarius*, has been associated with improved growth performance, feed conversion efficiency, and resistance against enteric pathogens in pigs [14]. A remarkable characteristic of *Ligilactobacillus* strains is their ability to produce antimicrobial compounds, including lantibiotics [15]. Nisin, a well-studied lantibiotic produced by different microbial species, has gained regulatory approval as a food preservative due to its broad-spectrum antimicrobial activity, stability, and safety profile [16].

Notably, numerous strains of *L. salivarius* have achieved recognition as Generally Recognized as Safe (GRAS) or have been granted the Qualified Presumption of Safety (QPS) status, further emphasizing their safety profile. The European Food Safety Authority (EFSA) provides guidelines and regulations for the evaluation of microbial feed additives. *Ligilactobacillus* strains, including *L. salivarius*, have been extensively studied and evaluated under the QPS framework, confirming their safety for intended applications [17]. However, it is important to recognize that the biosafety of novel candidate strains proposed for use as probiotics cannot be assumed or generalized. Thorough evaluation and careful assessment are necessary to ensure their safety and effectiveness [18]. Accordingly, the utilization of whole-genome sequencing (WGS) and subsequent in silico genome analysis based on WGS data can be an efficient approach to thoroughly assess the safety and functionality of the microorganisms being evaluated [19].

*L. salivarius* P1CEA3, a strain isolated from the GIT of pigs, has been previously identified as a bacteriocin-producing strain with a broad and strong antimicrobial activity against different pathogens including *Streptococcus suis*, other Gram-positive bacteria and *Escherichia coli*. The antimicrobial activity of *L. salivarius* P1CEA3 is mainly due to the production of the lantibiotic nisin S, the first fully characterized nisin variant produced by *L. salivarius* [20], which is a bacterial species recognized for its safety and probiotic potential. The present work aims to study the identification of probiotic and safety traits from a comprehensive genome-based in silico analysis of *L. salivarius* P1CEA3, and its potential in promoting the GI health in pigs.

## 2. Materials and Methods

### 2.1. Bacterial Strain, DNA Isolation and Genome Sequencing

*Ligilactobacillus salivarius* P1CEA3 was isolated from the GIT of slaughtered pigs as previously described [20]. *L. salivarius* P1CEA3 was grown overnight at 37 °C in tryptic soy broth (TSB) (Oxoid Ltd., Basingstoke, UK) under anaerobic conditions using anaerobic jars with an AnaeroGen 3.5 l pack (Oxoid). Total genomic DNA was extracted by using the DNeasy Blood & Tissue Kit (Qiagen, Hilden, Germany). The DNA was quantified in a Qubit fluorometer (Invitrogen, Thermo Fisher Scientific, Waltham, MA, USA), and its quality was confirmed by agarose gel electrophoresis in 0.8% (*w*/*v*) agarose (Condalab, Madrid, Spain) gel, which was visualized with a ChemiDoc Imaging System (BioRad, Hercules, CA, USA).

Whole-genome sequencing (WGS) of the purified DNA was performed by Illumina and Oxford Nanopore Technologies (ONT) at the SeqCenter (Pittsburgh, PA, USA). Sequencing quality and adapter trimming was performed with bcl2fastq v.2.20.0.445 and porechop v.0.2.3_seqan2.1.1 for Illumina and ONT sequencing, respectively. Read count statistics were recorded. Hybrid assembly with Illumina and ONT reads was performed with Unicycler v.0.4.8 [21]. The quality of the assembled sequences was assessed using the QUAST v.5.0.2 tool [22]. Assembly annotation was performed with Prokka v.1.14.5 [23]. The resulting DNA sequences were obtained in FASTA format. Unless otherwise stated, the following bioinformatics analyses were performed from the assembled genome FASTA sequence file as the input file.

### 2.2. Genome Features of L. salivarius P1CEA3

Bacterial species identification was performed by SpeciesFinder v.2.0. (https://cge.food.dtu.dk/services/SpeciesFinder/, accessed on 13 September 2022) [24], which predicts bacterial species by using the complete 16S rDNA sequence, and by KmerFinder v.3.2 (https://cge.cbs.dtu.dk/services/KmerFinder/, accessed on 13 September 2022), which is a tool that predicts bacterial identity using a fast k-mer algorithm based on the number of concurrent k-mers between the query genome and those genomes in the database. The Bioinformatics Application for Navigating de novo Assembly Graphs Easily (Bandage) software (https://rrwick.github.io/Bandage/, accessed on accessed on 9 May 2023) was used for the interactive visualization of the assembled genome. The DNAPlotter of The Artemis Software v18.0.1 (https://www.sanger.ac.uk/tool/dnaplotter/, accessed on 9 May 2023) [25] and the Proksee tool (https://proksee.ca/, accessed on 18 January 2023) [26] were also used for the generation of a graphical representation of the *L. salivarius* P1CEA3 genome. Coding DNA sequences (CDSs) were predicted and annotated using the Rapid Annotation Subsystem Technology (RAST) online server (http://rast.nmpdr.org/, accessed on 15 May 2023) [27] as well as the NCBI Submission Portal using Prokaryotic Genome Annotation Pipeline (NCBI-PGAP) [28]. Both portals were used to determine different features of the annotated whole genome sequence of *L. salivarius* P1CEA3 such as GC percentage, coding and non-coding proteins, RNAs and pseudogenes.

### 2.3. Comparison of Plasmids of L. salivarius P1CEA3 with Similar Plasmids

A BLASTn (NCBI) (https://blast.ncbi.nlm.nih.gov/Blast.cgi/, accessed on 16 May 2023) [29] of the different plasmid sequences of *L. salivarius* P1CEA3 as query was performed to find the existence of homologous plasmids in other LAB. The BLAST Ring Image Generator (BRIG) program (https://brig.sourceforge.net//, accessed on 16 May 2023) [30] was used for multiple *L. salivarius* megaplasmid comparisons with pMP1CEA3 as the refence sequence, including an upper identity threshold of 70% and lower identity threshold of 50%.

### 2.4. Bacteriocins and Secondary Metabolites

Bacteriocins and secondary metabolites gene clusters in the *L. salivarius* P1CEA3 genome were predicted by using the online web tools BAGEL v.4.0 (http://bagel4.molgenrug.nl/, accessed on 22 September 2022) [31], Antibiotics and Secondary Metabolite Analysis Shell (antiSMASH) (https://antismash.secondarymetabolites.org/, accessed on 22 September 2022) [32,33], Prediction Informatics for Secondary Metabolomes (PRISM 4) (http://magarveylab.ca/prism/, accessed on 9 May 2023) [34], and the SnapGene v.7.0.3 software (GSL Biotech, San Diego, CA, USA). BLASTp (NCBI) (https://blast.ncbi.nlm.nih.gov/Blast.cgi/, accessed on 16 May 2023) [29] and UniProt (https://www.uniprot.org//, accessed on 16 May 2023) [35] databases were used to confirm the identity of the encoded protein sequences of *L. salivarius* P1CEA3.

### 2.5. Transferable Antibiotic Resistances

The Proksee v1.0.0a6 web server (which uses The Comprehensive Antibiotic Resistance Database (CARD) Resistance Gene Identifier (RGI) [36]), the ResFinder tool v.4.1. database (https://cge.cbs.dtu.dk/services/ResFinder/, accessed on 18 January 2023) [37] and the KmerResistance v.2.2 web server (https://cge.food.dtu.dk/services/KmerResistance/, accessed on 18 January 2023) [38] were used for the in silico identification of genes that mediate antimicrobial resistances in the *L. salivarius* P1CEA3 genome.

The antibiotic resistance of *L. salivarius* P1CEA3 was also performed by using a phenotypic antibiotic resistance assay, as previously described [39]. The minimum inhibitory concentration (MIC) of *L. salivarius* P1CEA3 was determined by a broth microdilution test [40] for the following antibiotics at different concentrations: ampicillin (0.25–16 μg/mL), vancomycin (1–64 μg/mL), gentamicin (0.5–32 μg/mL), kanamycin (2–128 μg/mL), streptomycin (1–64 μg/mL), erythromycin (0.25–16 μg/mL), clindamycin (0.25–16 μg/mL), tetracycline (0.5–32 μg/mL), and chloramphenicol (1–64 μg/mL). MICs were established as the lowest antibiotic concentration inhibiting bacterial growth and interpreted according to the cut-off values adopted by the EFSA Panel on Additives and Products or Substances used in Animal Feed (FEEDAP) in relation to the “Guidance on the characterization of microorganisms used as feed additives or as production organisms” [17]. *Enterococcus faecalis* CECT 795 and *Staphylococcus aureus* CECT 794 were used as the control microorganisms.

### 2.6. Virulence and Pathogenicity

The PathogenFinder v.1.1 web server (https://cge.food.dtu.dk/services/PathogenFinder/, accessed on 18 January 2023) [41] was used to assess the pathogenicity of *L. salivarius* P1CEA3 on human health.

### 2.7. Mobile Genetic Elements (MGE): Insertion Sequences (IS), Genomic Islands (GI) and Prophages

Different MGEs were searched within the *L. salivarius* P1CEA3 genome using mobileOG-db (beatrix-1.6) [42] through the Proksee v1.0.0a6 web server program and the MobileElementFinder v.1.0.3 (https://cge.food.dtu.dk/services/MobileElementFinder/, accessed on 18 January 2023) [43] tool. The ISfinder database (https://www-is.biotoul.fr/index.php, accessed on 16 May 2023) [44] was used for the identification of insertion sequences (IS). For the prediction of genomic islands (GI), the IslandViewer 4 (http://www.pathogenomics.sfu.ca/islandviewer/, accessed on 16 May 2023) [45] server was used. The prediction methods used with this program were IslandPath-DIMOB [46], which facilitates the identification of prokaryotic GI including atypical sequence composition or the presence of genes associated with MGE, and SIGI-HMM [46], which predicts prokaryotic GI based on sequence composition and also employs a Hidden Markov Model (HMM) for the assessment of codon usage patterns in the identification of potential GIs. For the identification of prophage sequences in the *L. salivarius* P1CEA3 genome, the PHAge Search Tool—Enhanced Release (PHASTER) (https://phaster.ca/, accessed on 16 May 2023) [47] and the Prophage Hunter (https://prohunter.genomics.cn, accessed on 16 May 2023) [48] web servers were used. The Phigaro v.2.3.0 tool [49] of the Proksee v1.0.0a6 web server was also used for the same purpose.

### 2.8. CRISPR/CRISPR-Cas Systems

The CRISPRCasFinder (https://crisprcas.i2bc.paris-saclay.fr/CrisprCasFinder/Index, accessed on 18 May 2023) [50] and the CRISPRCasTyper (https://cctyper.crispr.dk/, accessed on 18 May 2023) [51] online programs were used for identification in the *L. salivarius* P1CEA3 genome of Clustered Regularly Interspaced Short Palindromic Repeats (CRISPR) and CRISPR-associated genes (*cas*).

### 2.9. Production of Biogenic Amines (BA) and Hemolytic and Gelatinase Activities

The in silico detection of genes responsible for the production of the BA histamine, tyramine, cadaverine and putrescine, including the genes encoding histidine decarboxylase, tyrosine decarboxylase, lysine decarboxylase, ornithine decarboxylase, phenylalanine decarboxylase, N-carbamoylputrescine amidase, and L-lysine decarboxylase [52], was performed by manual search through the functional annotation of the *L. salivarius* P1CEA3 genome in the NCBI-PGAP platform.

Putative genes involved in the synthesis of hemolysin and gelatinase by *L. salivarius* P1CEA3 were also sought manually. In addition, the putative hemolytic and gelatinase activities by *L. salivarius* P1CEA3 were also evaluated in vitro as previously described [53]. Hemolysin production was determined by growing the strain in TSB broth in anaerobiosis at 37 °C overnight, and then it was streaked on horse blood agar plates (BioMérieux), which were incubated in anaerobiosis at 37 °C for 24 h. The β-hemolysis was revealed by the presence of clear zones of hydrolysis around the colonies. For the production of gelatinase by *L. salivarius* P1CEA3, the culture was grown in anaerobiosis at 37 °C overnight and streaked onto Todd–Hewitt (Oxoid) agar plates (1.5%, *w*/*v*) supplemented with 30 g of gelatin per liter, and the culture was incubated anaerobically at 37 °C for 16 h. Subsequently, the plate was placed at 4 °C for 5 h before inspecting for the presence of turbid zones (indicating protein hydrolysis) surrounding the colonies. For both assessments, *E. faecalis* P4 was employed as positive control and *L. lactis* subsp. *lactis* BB24 was employed as negative control.

### 2.10. Probiotic Associated Traits

Genes related to probiotic functions were manually searched from the functional annotation of the *L. salivarius* P1CEA3 genome in the NCBI-PGAP and RAST platforms. Additionally, gene clusters related to probiotic traits were also searched using the NCBI-PGAP annotation and the SnapGene v.7.0.3 software. Amongst the probiotic traits analyzed, emphasis was placed on those related to adhesion and aggregation, vitamin biosynthesis, amino acids metabolism, antimicrobial activity, lactic acid production, and stress and host GIT adaptations. Other gene clusters and/or genes searched were those involved in the production of exopolysaccharides (EPS), surface adhesins, mucus-binding proteins (Mub), SecA2/SecY2 auxiliary translocation system, choloylglycine hydrolase, and L-lactate or/and D-lactate dehydrogenases.

## 3. Results and Discussion

### 3.1. Features Associated to the L. salivarius P1CEA3 Genome

**Figure 1:**
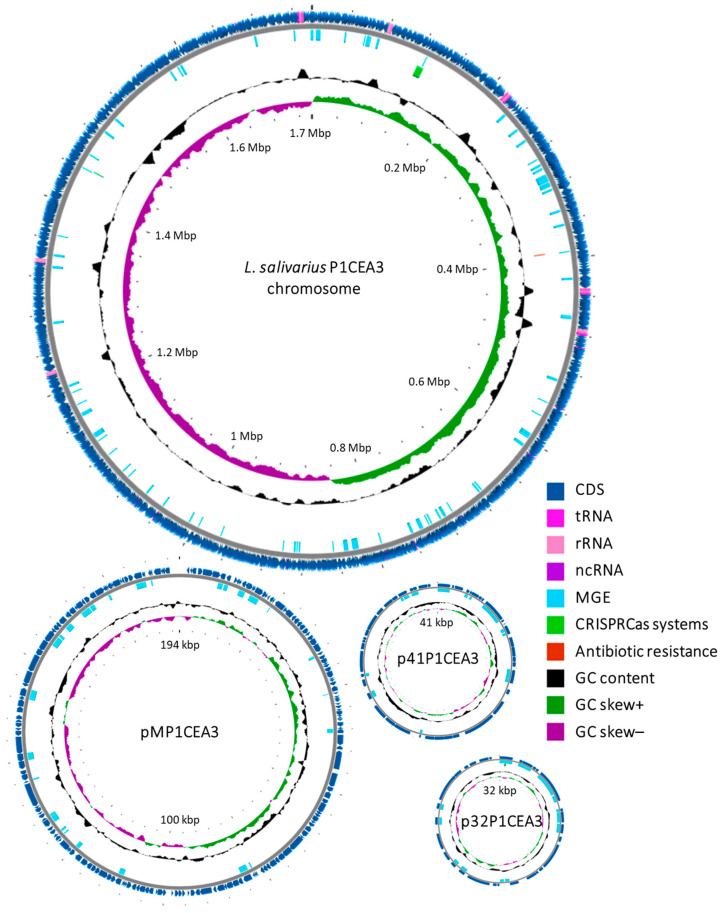
Chromosome and plasmids map of *L. salivarius* P1CEA3 created using Proksee v1.0.0a6 server (not to scale). This map shows the spread of CDS, tRNA, rRNA, ncRNA, MGE (mobile genetic elements), CRISPR-Cas systems, antibiotic resistance, and the GC content shift.

The hybrid assembly of the genome sequence of *L. salivarius* P1CEA3 with the Illumina and Oxford Nanopore Technologies (ONT) reads confirmed a genome of 2,007,767 bp, which was distributed in four circular contigs including a chromosome of 1,739,667 bp, a megaplasmid of 194,140 bp (pMP1CEA3) and two plasmids of 41,764 bp (p41P1CEA3) and 32,196 bp (p32P1CEA3) (Table 1). The visualization of the *L. salivarius* P1CEA3 annotated genome by using the Bandage, DNAPlotter v18.0.1 and Proksee v1.0.0a6 software tools confirmed the existence of four circular contigs (Figure 1). The overall GC content of the chromosome (32.5%) was similar to that of the megaplasmid pMP1CEA3 (31.5%) but lower than those of the plasmids p41P1CEA3 and p32P1CEA3 (39.5% and 38%, respectively) (Table 1), suggesting that these plasmids were most likely acquired via horizontal transfer from other species [54]. The species prediction made by the SpeciesFinder and KmerFinder web servers confirmed the identification of the sequenced strain as *L. salivarius*, which is an abundant species in the pig’s gut microbiota [55].

From the annotated genome sequence of *L. salivarius* P1CEA3 in the NCBI-PGAP (https://www.ncbi.nlm.nih.gov/genome/browse/, accessed on 2 August 2023), the *L. salivarius* P1CEA3 genome was shown to encode 2018 genes, 1882 protein-coding genes, 33 pseudogenes and 103 non-coding genes (Table 1), being these numbers close to the median gene counts, pseudogenes and non-coding genes of 30 complete genomes of *L. salivarius* deposited in the NCBI database (https://www.ncbi.nlm.nih.gov/genome/browse/, accessed on 2 August 2023).

### 3.2. Comparison of the L. salivarius P1CEA3 Plasmids with Other Plasmids

A BLASTn search against the NCBI database suggested similarities between the *L. salivarius* P1CEA3 plasmids with others found in different bacteria from the *Lactobacillaceae* family. By using a lower identity threshold set at 95% and a lower query cover set at 29%, the megaplasmid pMP1CEA3 was identified only in *L. salivarius* strains, whereas plasmid p41P1CEA3 was predominantly detected in *L. salivarius* but also in other bacterial species such as *Limosilactobacillus portuensis*, *Lactiplantibacillus plantarum*, *Lactobacillus gasseri*, and *Lactobacillus paragasseri*. Surprisingly, plasmid p32P1CEA3 was not identified in other *L. salivarius*, but it was present in *Limosilactobacillus gastricus*, *Lactobacillus paracasei*, *Lentilactobacillus hilgardii*, *Limosilactobacillus fermentum*, *Furfurilactobacillus rossiae*, *Lentilactobacillus parabuchneri* and *Lactiplantibacillus paraplantarum*. This suggests a horizontal plasmid transfer of p41P1CEA3 and p32P1CEA3 between different *Lactobacillus* species [56].

**Figure 2:**
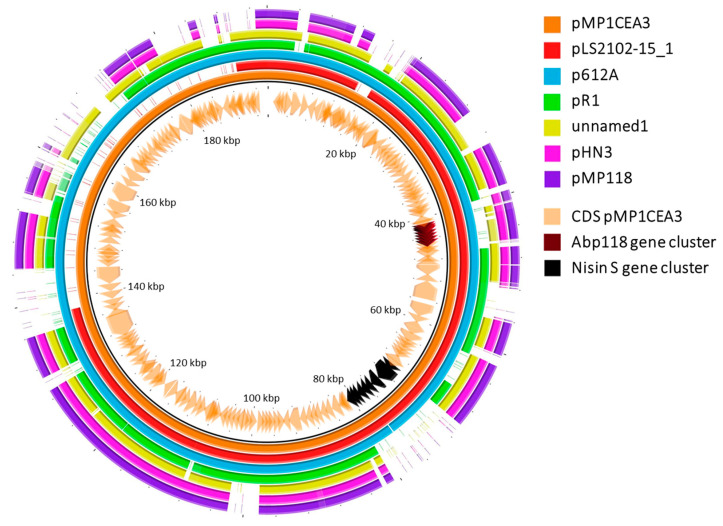
Comparison of megaplasmids of different *L. salivarius*. A BLAST atlas diagram of seven megaplasmids of *L. salivarius* was generated using BLAST Ring Image Generator (BRIG), employing pMP1CEA3 as the reference replicon (the inner orange ring). Working outwards from pMP1CEA3, the next six rings represent query plasmids of the *L. salivarius* strains named as pLS2102-15_1, p612A, pR1, unnamed1, pHN3 and pMP118. Regions of diversity between the megaplasmids are shown in light colors (less than 70% identity) and white color (less than 50% identity). The CDS of pMP1CEA3 was projected inside the black ring backbone and outside the kilobase pair (kbp) ruler at the center of the figure. Gene clusters for bacteriocins Abp118 and nisin S are shown within the CDS in garnet and black colors, respectively.

Furthermore, pMP1CEA3 was compared with other six megaplasmids identified in other *L. salivarius* strains with a high percent identity. Of interest were the differences observed in the gene clusters involved in the synthesis and secretion of the Abp118 and nisin S bacteriocins encoded in pMP1CEA3 compared to the other megaplasmids (Figure 2). The Abp118 gene cluster was present with high percent identity in pLS2102-15_1 and p612A. However, in pHN3, pMP118 and unnamed1 this region showed a lower percent identity, and in pR1, this gene cluster was absent. Importantly, the absence of the nisin S gene cluster was observed in four of the compared megaplasmids but not in two of them (pLS2102-15_1 and p612A). Differences in size and other genomic attributes (Table S1) had been also observed between circular megaplasmids of different strains of *L. salivarius* [57].

### 3.3. Bacteriocins and Secondary Metabolites

The assembled *L. salivarius* P1CEA3 genome, evaluated with the BAGEL v.4.0 and antiSMASH servers, indicated the existence of two distinct bacteriocin gene clusters in pMP1CEA3, one encoding the class II bacteriocins salivaricin B (SalB) and Abp118 (Abp118α and Abp118β), previously characterized in *L. salivarius* M6 and *L. salivarius* UCC118, respectively [58,59], and a second gene cluster encoding the lantibiotic nisin S [20]. The arrangement of genes within the nisin S gene cluster of pMP1CEA3 was *nssABTCRKFEG*, lacking the genes encoding a specific peptidase and an immunity protein, as compared to the nisin A gene cluster of *Lactococcus lactis* [20]. Previous results also demonstrated that although the *abp118* structural genes of *L. salivarius* P1CEA3 were functional, deletions and mutations in genes related to its transport (*abpT* and *abpD*) and regulation (*abpK*) were preventing the synthesis and secretion of Abp118. Furthermore, colony MALDI-TOF MS determinations and targeted proteomics combined with massive peptide analysis (LC-MS/MS) confirmed the presence of nisin S but not of SalB and Abp118 in the purified supernatants of *L. salivarius* P1CEA3 [20].

**Figure 3:**
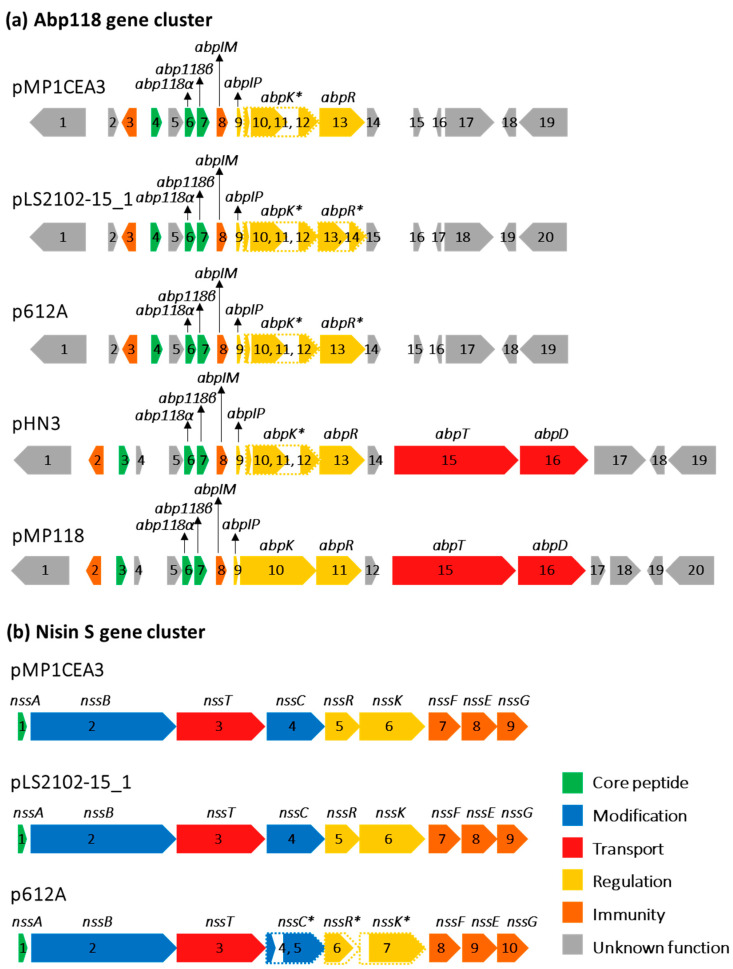
(**a**) Abp118 and (**b**) nisin S bacteriocin gene clusters in pMP1CEA3 from *L. salivarius* P1CEA3 as compared to those in megaplasmids pLS2102-15_1, p612A, pHN3 and pMP118. BAGEL v.4.0 and SnapGene v.7.0.3 programs were used as templates to generate the diagram. ORFs are indicated by arrows and numbers, and gene identity and color denote those with known functions. ORFs marked with a dashed arrow and their gene identity with an asterisk indicate putative non-functional ORFs.

BAGEL v.4.0 and SnapGene v.7.0.3 programs were used for a deeper comparison of the two bacteriocin gene clusters present in the *L. salivarius* megaplasmids under evaluation (Figure 3). The Abp118 gene cluster of pMP1CEA3 was similar to that in p612A with a truncated *abpK* regulator and the absence of the *abpT* and *abpD* transport genes. In pLS2102-15_1, the *abpT* and *abpD* transport genes were absent, and the *abpK* and *abpR* regulators were both truncated. In pHN3, the Abp118 gene cluster harbored the *abpT* and *abpD* transport genes, but the *abpK* regulator gene was truncated, as it occurs in pMP1CEA3, p612A and pLS2102-15_1. Importantly, the Abp118 gene cluster in pMP118 held all genes considered essential for the production, regulation and secretion of Abp118 (Figure 3a). From the results observed, the production and secretion of Abp118 by *L. salivarius* 2102-15 (pLS2102-15_1) and *L. salivarius* AR612 (p612A) would need to be confirmed.

A complete nisin S gene cluster was also identified in pLS2102-15_1 of *L. salivarius* 2102-15. The p612A of *L. salivarius* AR612 encoded the nisin S gene cluster, but further analysis of this region with the SnapGene v.7.0.3 software led to find a truncated *nssC* and deletions in *nssR* and *nssK* (Figure 3b). Accordingly, since nisin S production by *L. salivarius* P1CEA3 may be considered a probiotic trait, the potential role of this microorganism as a probiotic in animal production is reinforced [20,60].

Secondary metabolites identified through genome mining encompass a variety of small organic molecules with potent and diverse biological functions. The use of both the antiSMASH and PRISM 4 servers has suggested the presence, in the *L. salivarius* P1CEA3 chromosome, of a biosynthetic type III polyketide synthase (T3PKS) gene cluster encoding a hydroxymethylglutaryl-CoA synthase, which is a monomeric unit featuring a consistent integration of secondary metabolites. Upstream and downstream of the hydroxymethylglutaryl-CoA synthase gene, additional biosynthetic genes, regulatory genes, and other genes were determined (Figure S1). Bacterial secondary metabolism produces a rich source of bacterial compounds, some of which regulate the intestinal microecology and maintain the health of the host. As is known, T3PKS are small proteins associated with the biosynthesis of polyketides, natural metabolites that comprise the basic chemical structure of compounds including polyethers, macrolides, quinones, tetracycles and other substances, and with potential applications as anti-infective, anti-tumor and immunosuppressive agents. It has been suggested that T3PKS could potentially be associated with bacterial viability and antibacterial activity of the bacterial producers in the intestinal environment [61,62].

### 3.4. Transferable Antibiotic Resistances

Bacterial antibiotic resistance is a substantial biosafety issue, affecting human and animal health as well as the safety of the food and environment. While intrinsic antibiotic resistance encoded in bacterial genomes is not the primary concern, the existence of mobile antibiotic resistance genes represents a severe threat as they can disseminate to other bacteria through the mechanism of horizontal gene transfer [63].

Different bioinformatic tools were used to search for antibiotic resistance genes in the *L. salivarius* P1CEA3 genome. The Proksee v1.0.0a6 web server (which uses CARD) identified a vancomycin resistance gene cluster chromosomally encoded in *L. salivarius* P1CEA3, although *L. salivarius* are known to be intrinsically resistant to vancomycin [17,64]. Moreover, the BLASTn search performed against Resfinder v.4.1 and KmerResistance v.2.2 servers confirmed the absence of other transferable and acquired antibiotic resistances in the *L. salivarius* P1CEA3 genome.

To confirm these genomic results, a phenotypic assay of *L. salivarius* P1CEA3 resistance to different antibiotics was performed. The MICs of the evaluated antibiotics against *L. salivarius* P1CEA3 were in the range of the breakpoints reported by EFSA [17], being this microorganism sensitive to all antibiotics tested except vancomycin, to which the strain was resistant. *L. salivarius* P1CEA3 was sensitive to ampicillin (MIC: 2 μg/mL), gentamicin (MIC: 16 μg/mL), kanamycin (MIC: 64 μg/mL), streptomycin (MIC: 64 μg/mL), erythromycin (MIC: 0.5 μg/mL), clindamycin (MIC: 0.5 μg/mL), tetracycline (MIC: 1 μg/mL) and chloramphenicol (MIC: 4 μg/mL). Therefore, the genomic and phenotypic antibiotic resistance assays performed confirmed *L. salivarius* P1CEA3 as a non-resistant antibiotic strain.

### 3.5. Virulence and Pathogenicity

For the successful initiation of an infection, virulence factors are crucial in the processes of colonization, invasion, and onset of pathological alterations [19,65]. The evaluation of the *L. salivarius* P1CEA3 genome with the PathogenFinder v.1.1 server predicted this microorganism as a non-human pathogen.

### 3.6. Mobile Genetic Elements (MGE)

Plasmids, insertion sequences (ISs), genomic islands (GIs) and prophages are different types of Mobile Genetic Elements (MGEs) whose presence was predicted in the *L. salivarius* P1CEA3 genome. As described previously, the hybrid assembly of the *L. salivarius* P1CEA3 genome confirmed the presence of four circular contigs, including a megaplasmid (pMP1CEA3) and two more plasmids p41P1CEA3 and p32P1CEA3 (Table 1 and Figure 1).

Different MGEs searched by the mobileOG-db (beatrix-1.6) program through the Proksee v1.0.0a6 tool were identified in the *L. salivarius* P1CEA3 genome (Figure 1), which could mediate processes such as integration/excision, replication/recombination/repair, stability/defense, or the transfer of bacterial MGE and phages [42,66]. The ISfinder database was used to search for IS. Only matches showing a score greater than 1,000 and an E-value 0 were considered. There were no IS matching this criterion in the chromosome and p41P1CEA3. However, five IS6 families were found in pMP1CEA3 and one IS3 family was found in p32P1CEA3 (Table S2). ISs are small pieces of DNA that move within or between genomes, generally encode a transposase, are the smallest and most abundant autonomous transposable elements (TE), and are players in shaping their host genomes [66]. GIs, as determined by using the IslandViewer 4 (including IslandPath-DIMOB and SIGI-HMM prediction methods), were also predicted in the chromosome and pMP1CEA3 of *L. salivarius* P1CEA3 (Table S3). GIs are identified as specific DNA segments found among closely related strains, and their formation is believed to play a role in the diversification and adaptation of microorganisms, exerting a substantial influence on genome evolution and plasticity [67].

No prophages were found in plasmids of *L. salivarius* P1CEA3 as predicted by using the PHASTER, Prophage Hunter and Phigaro (Proksee v1.0.0a6) tools. However, PHASTER and Prophage Hunter tools identified a prophage in the *L. salivarius* P1CEA3 chromosome with PHAGE_Lactob_Sha1_NC_019489(7) as the most similar phage. Phigaro also identified in the *L. salivarius* P1CEA3 chromosome two prophage regions taxonomically similar to the *Siphoviridae* and *Myoviridae*/*Siphoviridae* families of double-stranded DNA viruses of bacteria and archaea, which are both not transposable. Prophages regulate bacterial gene expression and behavior across various bacterial species through mechanisms involving DNA rearrangements, transcription factors, and controlled bacterial lysis. This results in mutualistic relationships, fostering adaptively enhanced phage–host fitness under specific conditions [68].

### 3.7. CRISPR/CRISPR/Cas Systems

Clustered Regularly Interspaced Short Palindromic Repeat (CRISPR) and CRISPR/Cas systems provide adaptive immunity against phages, plasmids and other MGE in bacteria and archaea [69,70,71]. After analysis of the *L. salivarius* P1CEA3 genome with the CRISPRCasFinder and CRISPRCasTyper servers, only one CRISPR/Cas array manifested evidence of being functionally active (Figure S2). This array was located in the chromosome of *L. salivarius* P1CEA3 and was predicted as CRISPR-Cas type II-A. The CRISPR-associated genes (*cas*) were predicted as *cas9*_TypeII, *cas1*_TypeII and *cas2*_TypeI-II-III. This CRISPR/Cas array is also identified in Figure 1.

CRISPR-Cas is widespread among certain *Lactobacillus* species, but its presence varies between strains. Type II is the most prevalent variety throughout the genus, with II-A standing out as the dominant subtype. In lactobacilli, the Type II-A systems are naturally active in their native host in terms of expression and efficiently targeting invasive and genomic DNA. Together, these systems expand the potential targeting range of Cas9 and provide multiplexing potential in native hosts and heterologous genome editing purposes [72].

### 3.8. Biogenic Amines (BA), Hemolysin and Gelatinase Production

Decarboxylation pathways including, among others, biogenic amine (BA) production pathways, are widespread among LAB. These are mostly related to counteract acid resistance by regulating the intracellular pH and generating metabolic energy by creating a proton motive force and subsequently converting it into ATP. However, BA accumulation in substrates on which LAB grows is considered a health risk [52,73,74]. In this study, genes involved in the production of BA were absent in the *L. salivarius* P1CEA3 genome, except for the production of a putative ornithine decarboxylase (ODC), which might contribute to putrescine formation. Two different metabolic routes have been described in LAB for the biosynthesis of putrescine, the ODC pathway and the agmatine deiminase (AgDI) pathway, and the prevalence of both depends on the type of substrate on which the LAB grows [74]. However, results from the negative or very low production of BA by other *L. salivarius* strains [73] and the negative in vitro production of putrescine by *L. salivarius* CECT 5713, a human-derived strain with a putative gene cluster for the synthesis of putrescine [75], suggest that the putative BA genetic spotted in the *L. salivarius* P1CEA3 chromosome may be mainly related to counteract internal acid stress resistance.

The in silico manual search allowed the identification of two putative hemolysin family proteins in the genome of *L. salivarius* P1CEA3, but no unique gelatinase-related genes were found. Most important, the negative results for the in vitro hemolysis and gelatin hydrolysis as determined for *L. salivarius* P1CEA3 in this study suggest this microorganism would be absent of hemolytic and gelatinase activity. Both enzymatic activities are prevalent in other LAB, such as in some *E. faecalis* strains, which contribute to the severity of their infection [76].

### 3.9. Probiotic-Related Genes

NCBI-PGAP and RAST annotations of the assembled *L. salivarius* P1CEA3 genome facilitated the identification of genes associated to potential probiotic traits (Table S4). Additionally, gene clusters related to probiotic traits were also manually searched using the NCBI-PGAP annotation and the SnapGene v.7.0.3 software. Genes encoding most of the probiotic characteristics were located in the *L. salivarius* P1CEA3 chromosome, much less in the megaplasmid pMP1CEA3 including genes encoding the lantibiotic nisin S, a few genes in plasmid p41P1CEA3 and none in plasmid p32P1CEA3 (Table S4).

An important trait for a potential probiotic strain is the ability to adhere to the GIT of the host by mechanisms including, among others, the synthesis and production of exopolysaccharides (EPSs). EPSs, composed of long-chain polysaccharide chains, consist of sugar units, predominantly glucose, galactose, and rhamnose, in varying proportions. They are either released into the food matrix or stay affixed to the cell surface, forming capsular polysaccharides. EPSs have been proposed to yield several beneficial health effects, including lowering cholesterol, regulating intestinal immunity, anti-tumor and anti-inflammatory properties, and inhibiting pathogens by disrupting biofilms and suppressing adhesion [77]. As in *L. salivarius* UCC118 [78], the *L. salivarius* P1CEA3 genome shows two putative operons encoding the production of EPS. The first operon in the *L. salivarius* P1CEA3 chromosome encodes a transmembrane protein of the EpsG family of glycosyltransferases that may be involved in the production of EPS of the extracellular matrix during biofilm formation, and it is surrounded by genes generally present in other EPS gene clusters such as glycosyltransferases, hydrolases, sugar epimerases and transferases (Figure S3a).

The second EPS operon in the *L. salivarius* P1CEA3 chromosome encodes genes putatively involved in the synthesis of EPS such as transcriptional regulator (*epsA*), polymerization and chain length protein (*epsB*), tyrosine-protein kinase (*epsC*), protein-tyrosine phosphate phosphohydrolase (*epsD*), glycosyltransferase UDP-phosphate galactosephosphotransferase (*epsE*), various glycosyltransferases, mutase (*glf*), oligosaccharide translocase (*epsU*) and other biosynthetic proteins (Figure S3b). As in *L. salivarius* UCC118, this second operon constitutes a more complete processing unit than the first operon [78]. Of interest was the identification of the *rfb* gene cluster (*rfbACBD*) within the second EPS operon of *L. salivarius* P1CEA3. These genes are responsible of the biosynthesis of dTDP-l-rhamnose, a crucial precursor in the synthesis of the cell wall of many bacteria [79,80] and the production of cell wall polysaccharides and rhamnose-containing EPS, in *L. lactis* [81]. This rhamnose-rich EPS is known to activate the human immune system by increasing the expression of many interleukins and cytokines [82].

A number of bacterial surface adhesion proteins also interact with receptors in epithelial cells of the GIT of the host to facilitate bacterial binding. Probiotics and pathogens compete for the same cell receptors, aiming to attach to the gut lining of the host. Surface adhesion proteins have been suggested as mediators of bacterial adhesion [83]. Surface adhesins such as the *Lactobacillus* epithelium adhesins (LEA) mediate the binding of microbes to the host and thus might enhance bacterial colonization [84]. The production of two LEA-family epithelial adhesin proteins by *L. salivarius* P1CEA3 might contribute to adhesion for bacterial colonization (Table S4). Other surface adhesion proteins encoded in the genome of *L. salivarius* P1CEA3 were a mucus domain-binding protein (MucBP) which is unique to gut inhabiting LAB [83] and a fibronectin type III-domain binding protein (Table S4). A gene encoding a class A sortase protein (LPXTG specific) was also identified in the *L. salivarius* P1CEA3 genome (Table S4). Sortase A (SrtA) determines bacterial adherence and communication with the mucosal immune system, and it is an enzyme capable of anchoring a large number of proteins to the cell wall. *srtA* deletion mutants have demonstrated a decreased in vitro porcine mucin adhesion and, thus, gut retention of probiotic microbes in the GIT is enhanced due to sortase-dependent proteins [85].

Genes related to the synthesis of the B-group vitamins such as thiamin (vitamin B1), riboflavin (B2), pyridoxin (B6), biotin (B7) and folate (B11) were identified in *L. salivarius* P1CEA3. Interestingly, two genes for the biosynthesis of riboflavin were located in pMP1CEA3, one of them (ribulose-phosphate 3-epimerase) being the only copy in the genome. The B-group vitamins are crucial in swine production and overall animal health. These vitamins improve the intestinal health, and folate-producing probiotic strains could confer protection against inflammation and cancer [86,87]. The NCBI-PGAP and RAST annotations have also allowed the identification, in *L. salivarius* P1CEA3, of genes involved in the biosynthesis of amino acids such as threonine, tryptophan, methionine, lysine, cysteine and arginine (Table S4). However, genes for the biosynthesis of leucine and histidine were not found in the genome of the strain, which is not a surprising feature in lactobacilli [78].

*Lactobacillus* can produce L-lactate or D-lactate or a combination of both by L-lactate (L-LDH) or D-lactate (D-LDH) dehydrogenases, respectively [88]. *L. salivarius* P1CEA3 encoded two copies of L-LDH in the chromosome and three copies of D-LDH, two in the chromosome and another in pMP1CEA3 (Table S4). Genes encoding L-LDH and D-LDH were also identified in *L. salivarius* UCC118 [78]. The lactate generated by LAB lowers the pH within the GIT, thereby inhibiting the proliferation of potentially pathogenic bacteria [87,89]. Of interest is the misperception about the D-lactic production by probiotic bacteria being responsible for chronic conditions in humans and animals without conclusive evidence [90].

Genes encoding proteins related to tolerance to temperature, acid, pH, bile salts, osmotic and oxidative stress were also identified in the *L. salivarius* P1CEA3 genome (Table S4), suggesting a high level of stress adaptation and bacterial survival [91,92]. Genes encoding the molecular co-chaperones GroES and GroEL, involved in the refolding of many proteins, also supports the probiotic potential of *L. salivarius* P1CEA3 to minimize the impact of the high temperatures reached during the processing of animal feed [93]. Osmotic adaptation in *L. salivarius* P1CEA3 may be also enhanced due to the L-proline glycine betaine ABC transport system permease protein genes *proX* and *proV* identified in p41P1CEA3, while its oxidative stress tolerance may be stronger due to the presence of the NAD(P)/FAD-dependent oxidoreductase gene, which is identified in the same plasmid. A gene encoding a choloylglycine hydrolase family protein was also identified in the *L. salivarius* P1CEA3 genome, as well as a truncated form of the protein in pMP1CEA3. This choloylglycine hydrolase is highly homologous to that encoded by *L. salivarius* UCC118 [78]. The choloylglycine hydrolase (bile salt hydrolase) is an enzyme synthesized by the intestinal microbiota, and its role involves catalyzing the hydrolysis of amide bonds in conjugated bile acids, leading to the release of free amino acids. This enzyme serves mutualistic purposes, benefiting both microbes and hosts. For microbes, it offers bile detoxification and aids in gastrointestinal endurance, while for hosts, it contributes to reducing cholesterol levels [94].

A bifunctional acetaldehyde-CoA/alcohol dehydrogenase was also encoded by *L. salivarius* P1CEA3 in pMP1CEA3 as it occurs in pMP118 of *L. salivarius* UCC118. While this dehydrogenase may not be deemed essential for bacterial producers, it possesses the potential to enhance their redox-balancing capability, since it has the ability to catalyze the conversion of acetyl-CoA to ethanol through the formation of acetaldehyde [78]. Genes involved in the SecA2/SecY2 auxiliary translocation system were absent in the *L. salivarius* P1CEA3 genome. This system has been found responsible for secreting virulence factors and post-translationally modified glycoproteins. Although the SecA2/SecY2 protein secretion system, comprising genes *secA2*, *secY2*, three accessory secretory proteins genes (*asp1*, *asp2* and *asp3*), and various glycosylation genes, such as nucleotide sugar synthetase gene (*nss*) and glycosyltransferase gene (*gtf*), is present in most *L. salivarius* strains isolated from pigs and chickens, this system has been not found yet in human *L. salivarius* isolates [94]. The absence of the SeA2/SecY2 system in *L. salivarius* P1CEA3 suggests the convenience to perform a more intense comparative genomic analysis of the identified *L. salivarius* strains, focusing on its physiology and host adaptation.

This study reports the evaluation of safety and probiotic traits from a genome-based in silico analysis of *L. salivarius* P1CEA3, which is isolated from pigs and a producer of nisin S. The absence of transferable antibiotic resistance determinants and other virulence factors, as well as the identification of a number of probiotic traits, support the safety of the strain. While some of the identified probiotic characteristics need to be further assessed to confirm their expression, the results obtained strongly support the potential of *L. salivarius* P1CEA3 as a potential probiotic in pig production.

## Figures and Tables

**Table 1:** Genome features of *L. salivarius* P1CEA3.

Feature	Chromosome	pMP1CEA3	p41P1CEA3	p32P1CEA3
Replicon size (bp)	1,739,667	194,140	41,764	32,196
GC content (%)	32.5	31.5	39.5	38
Topology	Circular	Circular	Circular	Circular
% of genome size	86.6	9.7	2.1	1.6
Coding genes	1624	191	40	27
Pseudogenes	15	7	5	6
rRNA	22	0	0	0
tRNAs	77	0	0	0
ncRNAs	4	0	0	0

## Data Availability

The whole genome assembly of *L. salivarius* P1CEA3 is deposited in GenBank under the accession number CP116812-CP116815. The nucleotide sequences reported in this study have the GenBank accession numbers from CP116812 to CP116815. Data is contained within the article.

## Supplementary Materials

The following supporting information can be downloaded at https://www.mdpi.com/article/10.3390/foods13010107/s1, Figure S1: Secondary metabolite-producing region T3PKS in the *L. salivarius* P1CEA3 genome as determined with the antiSMASH web tool. The arrows and the colors indicate gene function; Figure S2: CRISPR/Cas system of *L. salivarius* P1CEA3 as determined by using the CRISPRCasTyper online server. Interference module in yellow, adaptation module in dark blue, accessory genes in pink, arrays with their associated subtype in black/white checkerboard, and unknown genes in gray. Cas genes with low-quality alignments are shown in parentheses around the name; and Figure S3: Biosynthetic genetic clusters for exopolysaccharides (EPS) production in the *L. salivarius* P1CEA3 chromosome cluster 1 (A), and the *L. salivarius* P1CEA3 chromosome cluster 2 (B); Table S1: Comparative features of some *L. salivarius* megaplasmids; Table S2: Insertion sequences (IS) in the *L. salivarius* P1CEA3 genome by using ISfinder database; Table S3: Genetic islands (GIs) in the *L. salivarius* P1CEA3 genome determined by using IslandViewer 4; Table S4: Probiotic characteristics based on *L. salivarius* P1CEA3 genome analysis.
