# Supplementary material for: Evaluation of Safety and Probiotic Traits from a Comprehensive Genome-Based In Silico Analysis of Ligilactobacillus salivarius P1CEA3, Isolated from Pigs and Producer of Nisin S
Source: Foods. 2023 Dec 28;13(1):107. doi: 10.3390/foods13010107 (PMC10778751; doi:10.3390/foods13010107)
Supplement: Supplementary file 1 [file foods-13-00107-s001.zip › foods-2752442-supplementary.pdf]

Table S1. Comparative features of some *L. salivarius* megaplasmids.

Plasmid	Strain	Size (bp)	GC content (%)	GenBank
pMP1CEA3	*L. salivarius* P1CEA3	194,140	31.5	CP116813.1
pLS2102-15_1	*L. salivarius* 2102-15	140,826	31.5	CP090412.1
p612A	*L. salivarius* AR612	191,673	31.5	CP123987.1
pR1	*L. salivarius* Ren	176,951	32	CP011404.1
unnamed1	*L. salivarius* BCRC 14759	405,494	32	CP024068.1
pHN3	*L. salivarius* CECT 5713	242,962	32	CP002037.1
pMP118	*L. salivarius* UCC118	242,436	32	CP000234.1

Table S2. Insertion sequences (IS) in the *L. salivarius* P1CEA3 genome by using ISfinder database.

Sequences producing significant alignments	IS Family	Origin	Score (bits)	E. value
**pMP1CEA3**				
IS1216E	IS6	*Enterococcus faecium*	1467	0.0
IS1216V	IS6	*Enterococcus* sp.	1435	0.0
IS1216	IS6	*Enterococcus hirae*	1390	0.0
ISS1W	IS6	*Lactococcus lactis*	1136	0.0
ISLmo19	IS6	*Listeria monocytogenes*	1021	0.0
**p32P1CEA3**				
IS153	IS3	*Lactobacillus sanfranciscensis*	1150	0.0

Table S3. Genetic islands (GI) in the of *L. salivarius* P1CEA3 genome determined by using IslandViewer 4.

**Start**	**End**	**Size (bp)**	**GI Prediction Method**
**Chromosome**			
42,164	46,381	4,217	SIGI-HMM
227,370	244,144	16,774	IslandPath-DIMOB
295,090	305,872	10,782	IslandPath-DIMOB
320,518	326,635	6,117	IslandPath-DIMOB
463,591	470,695	7,104	SIGI-HMM
916,732	940,983	24,251	IslandPath-DIMOB
1,485,169	1,499,686	14,517	IslandPath-DIMOB
1,543,942	1,557,755	13,813	IslandPath-DIMOB
1,641,885	1,652,470	10,585	IslandPath-DIMOB
**pMP1CEA3**			
120,001	125,978	5,977	SIGI-HMM

Table S4. Probiotic characteristics based on *L. salivarius* P1CEA3 genome analysis.

Gene Function	Size of the gene (bp)			
	Chromosome	pMP1CEA3	p41P1CEA3	p32P1CEA3
**Adhesion and aggregation**				
Exopolysaccharides (EPS) biosynthetic gene clusters				
Exopolysaccharide biosynthesis protein	810			
Glycosyltransferase family 1 protein	1107			
Manganese-dependent protein-tyrosine phosphatase	777			
Tyrosine-protein kinase EpsD	663/726			
Tyrosine-protein kinase transmembrane modulator EpsC	798			
LEA family epithelial adhesin protein	1755/1161			
Mucus-binding protein MucBP	1041			
Fibronectin-binding protein	1689			
Sortase A, LPXTG	621			
Enolase	1326			
Triosephosphate isomerase	756			
ATP synthase epsilon chain	420			
DNA-directed RNA polymerase subunit epsilon	216			
**Vitamin biosynthesis**				
**Thiamine**				
Xanthine/uracil/thiamine/ascorbate permease family protein	1314			
Uracil permease	1308			
HMP-PP hydrolase (pyridoxal phosphatase) Cof	807/822			
Hydroxyethylthiazole kinase				
Hydroxymethylpyrimidine kinase/phosphomethylpyrimidine kinase	795			
Hydroxymethylpyrimidine ABC transporter, ATPase component				
Hydroxymethylpyrimidine ABC transporter, substrate-binding component				
Hydroxymethylpyrimidine ABC transporter, transmembrane component				
Substrate-specific component ThiT of thiamin ECF transporter	558			
Substrate-specific component ThiW of predicted thiazole ECF transporter				

Thiamin pyrophosphokinase	663	
**Riboflavin**		
3,4-dihydroxy-2-butanone 4-phosphate synthase		
6,7-dimethyl-8-ribityllumazine synthase ATP phosphoribosyltransferase		
Diacylglycerol kinase	927/417	
5- amino-6-(5-phosphoribosylamino)uracil reductase		
Hypothetical protein YebC	729	
NADH dehydrogenase	645/1206	618
N-terminal domain of CinA protein/ C-terminal domain of CinA type S		
Orotidine 5'-phosphate decarboxylase	714	
Phosphoribosyl-AMP cyclohydrolase/Phosphoribosyl-ATP pyrophosphatase		
Riboflavin kinase/ FMN adenylyltransferase	957	
Riboflavin synthase eubacterial/eukaryotic		
Ribulose-phosphate 3-epimerase		648
Substrate-specific component RibU of riboflavin ECF transporter	597	
Transcription termination protein NusB	396	
tRNA pseudouridine synthase B	908	
**Pyridoxin**		
D-3-phosphoglycerate dehydrogenase	1185/ 957	
Hypothetical NagD-like phosphatase	774	
1-deoxy-D-xylulose 5-phosphate synthase	1734	
Pyridoxamine 5′-phosphate oxidase	408	
Pyridoxine biosynthesis glutamine amidotransferase, glutaminase subunit		
NAD-dependent glyceraldehyde-3-phosphate dehydrogenase	1013	
**Biotin**		
3-ketoacyl-CoA thiolase Acetyl-CoA acetyltransferase	675	
Acetoacetyl-CoA synthetase/Long-chain-fatty-acid–CoA ligase		
Adenosylmethionine-8-amino-7-oxononanoate aminotransferase		
ATPase component BioM of energizing module of biotin ECF transporter		
Biotin synthase		

Biotin--protein ligase/ Biotin operon repressor	966
Competence protein F homolog, phosphoribosyltransferase domain	
Substrate-specific component BioY of biotin ECF transporter	537
Biotin carboxyl carrier protein of acetyl-CoA carboxylase	450
Biotin carboxyl carrier protein of methylcrotonyl-CoA carboxylase	
Biotin carboxylase of acetyl-CoA carboxylase	1380
Biotin carboxylase of methylcrotonyl-CoA carboxylase	
**Folate**	
Dihydrofolate synthase/ Folylpolyglutamate synthase	1302
GTP cyclohydrolase I type 1	
Pantoate—beta-alanine ligase	
Aspartate 1-decarboxylase	
2-amino-4-hydroxy-6-hydroxymethyldihydropteridine	
Dihydroneopterin aldolase	
Cell division protein FtsH	2079
Hypoxanthine-guanine phosphoribosyltransferase	540
tRNA (Ile)-lisidine synthetase	1320
Para-aminobenzoate synthase, amidotransferase component	720
5-formyltetrahydrofolate cyclo-ligase	549
Dihydrofolate reductase	504
Thymidylate synthase	957
**Amino acids metabolism**	
**Threonine**	
Biosynthetic aromatic amino acid aminotransferase alpha	
Aspartokinase	1197
Homoserine dehydrogenase	1287
Aspartate aminotransferase	1119/1176/1182/1188
Aspartate-semialdehyde dehydrogenase	1092
Homoserine kinase	879
Threonine synthase	1488
**Tryptophan**	

Tryptophan ABC transporter substrate-binding protein	993
Tryptophanyl-tRNA synthetase	1020
Tryptophan synthase alpha chain	
Tryptophan synthase beta chain	
Phosphoribosylformimino-5-aminoimidazole carboxamide ribotide isomerase	
Para-aminobenzoate synthase, amidotransferase component	720
Isochorismatase	
**Methionine**	
Peptide methionine sulfoxide reductase	435/519
Cysteine beta-synthase	918
Cystathionine beta-lyase	
Cystathionine gamma-lyase	1143
S-adenosylmethionine synthetase	1191
Serine acetyltransferase	501
Homoserine O-succinyltransferase	807/852
Methionine ABC transporter ATP-binding protein	1062/1059
Homoserine kinase	879
Homoserine dehydrogenase	1287
5-methyltetrahydrofolate—homocysteine methyltransferase	
5,10-methylenetetrahydrofolate reductase	
O-acetylhomoserine sulfhydrylase	1299
SAM-dependent methlytransferase YrrT	717/1020/735/612
**Lysine**	
Aspartokinase	1197
Lysine-epsilon oxidase	
4-hydroxy-tetrahydrodipicolinate synthase	873
4-hydroxy-tetrahydrodipicolinate reductase	789
Aspartate-semialdehyde dehydrogenase	1092
Diaminopimelate decarboxylase	1293
N-acetyl-L,L-diaminopimelate deacetylase	1146

2,3,4,5-tetrahydropyridine-2,6-dicarboxylate N-acetyltransferase	705	
N-acetyl-L,L-diaminopimelate aminotransferase	1164	
**Cysteine**		
Cysteine synthase	924	
Thioredoxin	318/312/324	
Cysteinyl-tRNA synthetase	1413	
Serine acetyltransferase	501	
Sulfite reductase [NADPH] hemoprotein beta-component		
Sulfite reductase [NADPH] flavoprotein alpha-component		
**Arginine**		
N-acetyl-gamma-glutamyl-phosphate reductase		
N-acetylglutamate synthase	624	
Acetylglutamate kinase		
Argininosuccinate synthase	1212	
Argininosuccinate lyase	1383	
Arginine pathway regulatory protein ArgR	444/459	
**Production of lactic acid**		
D-lactate dehydrogenase	993/990	996
L-lactate dehydrogenase	969/909	
**Stress and host gastrointestinal tract (GIT) adaptation**		
**Temperature tolerance**		
Cold shock protein CspA	201	
Cold shock protein CspC		
Cold shock protein CspP		
Heat shock protein DnaJ	1134	
Heat shock protein DnaK	573	
Heat shock protein HtpX	888	
Heat shock protein Hsp33	891	
Heat shock protein GrpE	573	
Heat shock protein Hsp70		
Heat shock protein 60 family chaperone GroEL	1623	

Heat shock protein 60 family co-chaperone GroES	285	
S4-domain-containing heat shock protein	270	
Ribosome-associated heat shock protein Hsp15		
**Acid tolerance**		
ATP synthase subunit a	714	
ATP synthase subunit b	528	
ATP synthase subunit c	225	
ATP synthase alpha chain	1512	
ATP synthase beta chain	1407	
ATP synthase gamma chain	930	
ATP synthase epsilon chain	420	
ATP synthase delta chain	543	
Na+/H+ antiporter	1386	
PTS system, cellobiose-specific IIC component	1332	
ATP-dependent Clp protease ATP-binding subunit	2193/1227	
Glucose-6-phosphate isomerase	1353	
GTP pyrophosphokinase	606/2220/645	
Pyruvate kinase	1761	
**pH tolerance**		
Sodium:proton antiporter	1590	
Na+/H+ antiporter NhaC	1386	
Alkaline shock protein	423	
F0F1 ATP synthase subunit A	714	
F0F1 ATP synthase subunit B	528	
F0F1 ATP synthase subunit C	225	
F0F1 ATP synthase subunit alpha	1512	
F0F1 ATP synthase subunit beta	1407	
F0F1 ATP synthase subunit gamma	930	
F0F1 ATP synthase subunit epsilon	420	
**Bile salts tolerance**		
Choloylglycine hydrolase (bile salt hydrolase)	978	813

Glucosamine-6-phosphate deaminase	705		
CTP synthase	1599		
**Osmotic stress and oxidative tolerance**			
Glycine betaine/choline			
a. Choline binding protein A	3000/2343/2970/567		
b. L-proline glycine betaine binding ABC transporter protein ProX			1524
c. L-proline glycine betaine ABC transport system permease protein ProV			972
d. ABC transporter, permease protein	1194		
d. Transport protein	933/1830/933	1221/1200	
Manganese-dependent inorganic pyrophosphatase	921		
Catalase			
Thiol peroxidase	495		
NAD(P)/FAD-dependent oxidoreductase			1332
Glutathione peroxidase			
NADH peroxidase	1356/1347		
NADH oxidase			
Flavin-dependent oxidoreductase	1065		
Haloperoxidase	678		
Iron dependent peroxidase			
Thioredoxin	318/312/324		
Thioredoxin reductase (NADPH)	1026/936		
Pyruvate oxidase	1887/1809		
Dihydroorotate oxidase			
Dihydroorotate dehydrogenase	945		
Lead, cadmium, zinc and mercury transporting P-type ATPase	615/1284/1911/2154/1902		
Manganese transport protein	1566		
Peptide methionine sulfoxide reductase	435/519		

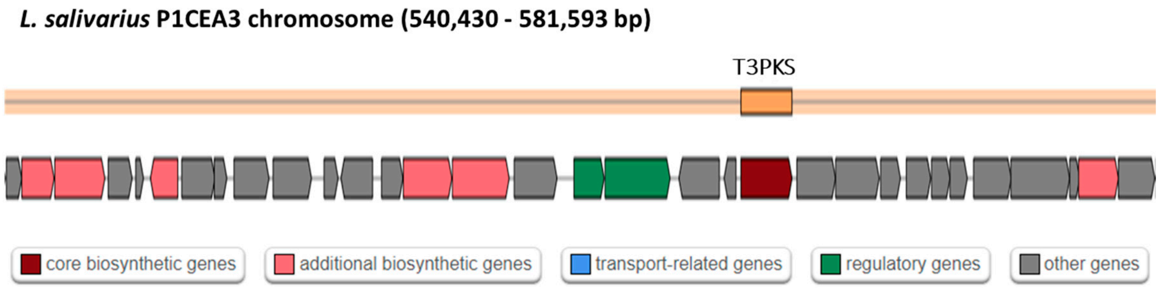

Figure S1. Secondary metabolite-producing region T3PKS in the *L. salivarius* P1CEA3 genome as determined with the antiSMASH web tool. The arrows and colors indicate gene function.

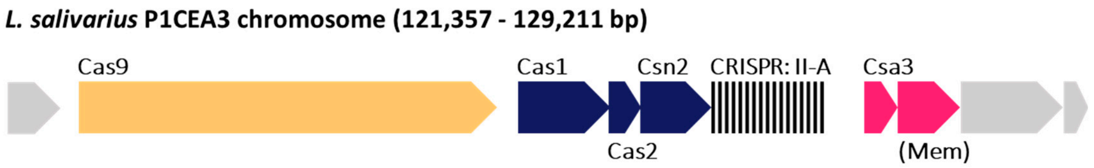

Figure S2. CRISPR/Cas system of *L. salivarius* P1CEA3 as determined by using the CRISPRCasTyper online server. Interference module in yellow, adaptation module in dark blue, accessory genes in pink, arrays with their associated subtype in black/white checkerboard, and unknown genes in grey. Cas genes with low-quality alignments are shown in parenthesis around the name.

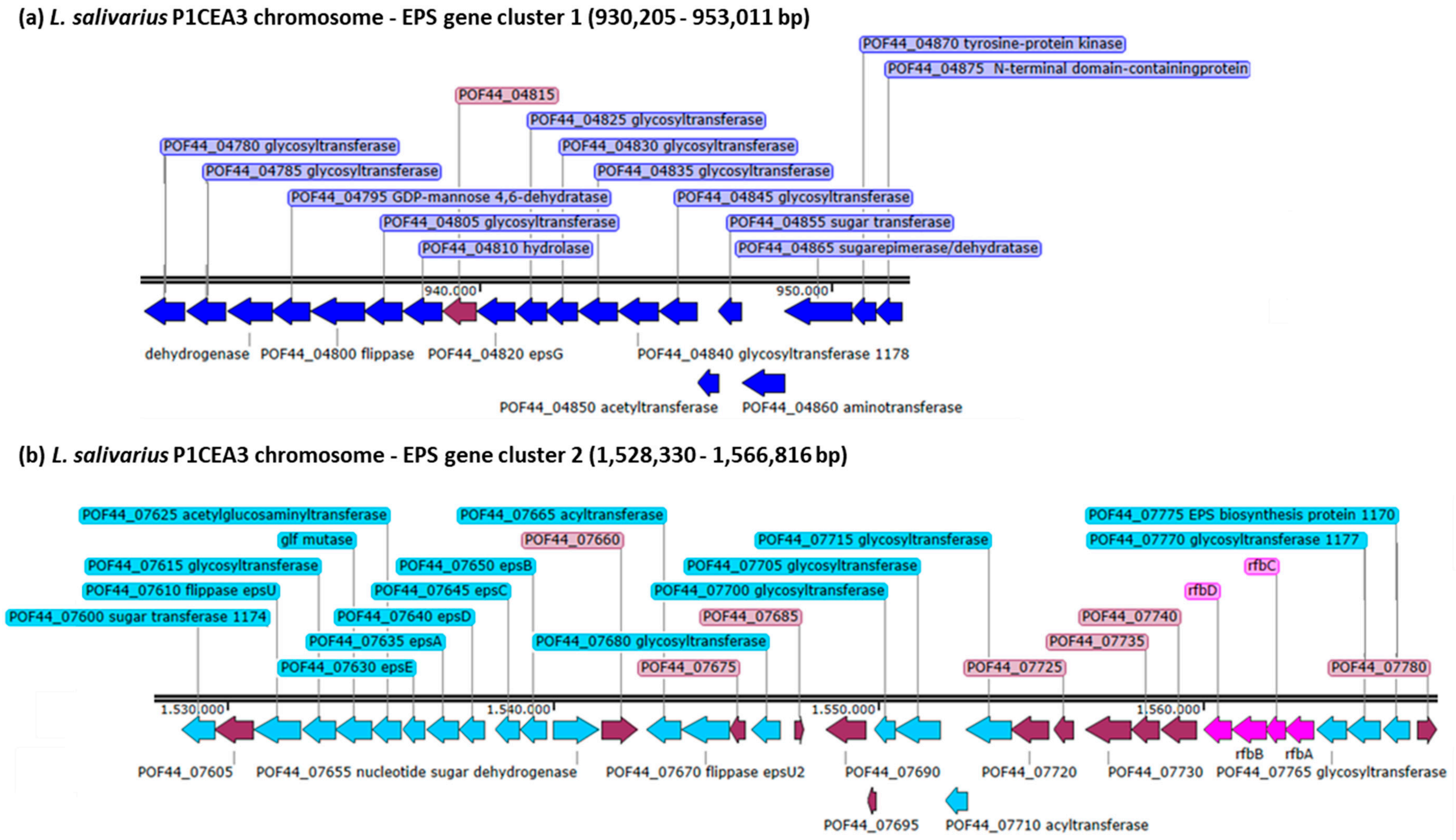

Figure S3. Biosynthetic genetic clusters for exopolysaccharide (EPS) production in (a) *L. salivarius* P1CEA3 chromosome cluster 1, and (b) *L. salivarius* P1CEA3 chromosome cluster 2.
